# Correction: Prevalence and associated factors for self-reported symptoms of dry eye among Thai school children during the COVID-19 outbreak

**DOI:** 10.1371/journal.pone.0314413

**Published:** 2024-11-20

**Authors:** Danai Tonkerdmongkol, Teera Poyomtip, Chotika Poolsanam, Akarapon Watcharapalakorn, Patarakorn Tawonkasiwattanakun

In the Methods section, there is an error in the second sentence of the first paragraph. The correct sentence is: The Research Ethics Review Committee for Research Involving Human Research Participants Group I at Chulalongkorn University (COA No. 208/2021) approved the study protocol on October 7, 2021.

## References

[1] Tonkerdmongkol D, Poyomtip T, Poolsanam C, Watcharapalakorn A, Tawonkasiwattanakun P (2023) Prevalence and associated factors for self-reported symptoms of dry eye among Thai school children during the COVID-19 outbreak. PLOS ONE 18(4): e0284928. 10.1371/journal.pone.0284928 37093868 PMC10124893

