# Supplementary material for: Prevalence and associated factors for self-reported symptoms of dry eye among Thai school children during the COVID-19 outbreak
Source: PLoS One. 2023 Apr 24;18(4):e0284928. doi: 10.1371/journal.pone.0284928 (PMC10124893; doi:10.1371/journal.pone.0284928)
Supplement: Attachment — Submitted filename: Response to Reviewers.docx [file pone.0284928.s002.docx]

**Journal Requirement**

1. Please ensure that your manuscript meets PLOS ONE's style requirements, including those for file naming. The PLOS ONE style templates can be found at 
https://journals.plos.org/plosone/s/file?id=wjVg/PLOSOne_formatting_sample_main_body.pdf 
https://journals.plos.org/plosone/s/file?id=ba62/PLOSOne_formatting_sample_title_authors_affiliations.pdf

*Thank you for your suggestion. In the current version, the manuscript was changed into the PLOS ONE style.*

2. Please amend your current ethics statement to address the following concerns:
a) Did participants provide their written or verbal informed consent to participate in this study?
b) If consent was verbal, please explain i) why written consent was not obtained, ii) how you documented participant consent, and iii) whether the ethics committees/IRB approved this consent procedure.

*In the current version, the ethics statement has been amended. This study is an online survey in which the informed consent was “click-if-you agree”. This method was applied due to the COVID-19 situation and the social-isolation policy that was still ongoing as shown in lines 93 – 94.*

3. You indicated that you had ethical approval for your study. In your Methods section, please ensure you have also stated whether you obtained consent from parents or guardians of the minors (participants below the age of 18 years) included in the study or whether the research ethics committee or IRB specifically waived the need for their consent.

*In the current version, it is indicated that we obtained the consent from parents or guardians.*

4. We suggest you thoroughly copyedit your manuscript for language usage, spelling, and grammar. If you do not know anyone who can help you do this, you may wish to consider employing a professional scientific editing service.

*The revised manuscript has been carefully reviewed by an experienced editor whose first language is English and who specializes in editing papers written by scientists whose native language is not English.*

5. Thank you for stating the following in the Acknowledgments Section of your manuscript:

“This work was financially supported by the 2021 Fund from the Faculty of Optometry, Ramkhamhaeng University.” However, funding information should not appear in the Acknowledgments section or other areas of your manuscript. We will only publish funding information present in the Funding Statement section of the online submission form. 
Please remove any funding-related text from the manuscript and let us know how you would like to update your Funding Statement.

*The funding statement form has been removed from the acknowledgments section.*

**Reviewer 1 Comments**

1. The Literature review sets the stage for the study questions, supported by updated and relevant studies. The literature review is based on relevant studies about self-reported symptoms and clinical examinations of young adults and youth, related to exposure-time to digital media. The authors should re-examine that the review relates to all variables examined, e.g., it lacks information regarding gender differences from previous studies.

*In the revised manuscript, the introduction has been reorganized by adding the information regarding the other factors of dry eye, i.e. gender, smoking, contact lens, and perceived stress, as shown in lines 76 – 79.*

2. The abstract points out that smoking and contact lens wear were not predisposing factors for dry eye, however the relevant groups were too small to have a significant statistical conclusion (not mentioned in the limitations section).

*Thank you for recommendation. We are truly in agreement with you about the limitation of the small proportion of contact lens wearing and smoking groups.*

*Therefore, we added the limitation regarding the small groups, as shown in lines 322 – 325.*

3. The researchers' assumption of dry eye prevalence greater than 50% should be further established, since all the formula calculations are based on this rate. This rate seems high according to the best of my knowledge of the medical literature, especially among younger populations.

*We agree with you that this rate may be high among a younger population, but we assumed 50% because:*

*1) During the first step of the project, we did not have the previous evidence regarding the dry eye symptoms in schoolchildren in the Thai population.*

*2) Changes in lifestyle due to the COVID-19 pandemic may prolong screen time in children, which may result in increased dry eye symptoms in children and adolescents. Therefore, we assumed 50% of prevalence to provide the highest number for the sample size.*

*Presently, there are several studies that show that the prevalence of dry eye is greater than 50% among university students,^1^ including Thailand.^2^ Moreover, the study in China indicated approximately 70% of dry eye among senior secondary school students during the COVID-19 pandemic.^3^ Thus, this rate is possible in this situation.*

4. The literature review leads to the study aims and the clear research questions but lacks a more specific hypothesis.

*In the revised manuscript, this section was re-written and combined with the previous paragraph*

*as shown in lines 80 – 83.*

5. the sampling process and the research population are well described. The research tools are well described, revealing quite high reliability, although they were originally established for adults. Authors should add information regarding translation validation.

*Thank you for your recommendation. In the current version, we have added the information regarding the translation process, as shown* in *lines 122 – 129.*

6. The authors provided rich information accompanied by detailed tables and plots, demonstrating the higher prevalence among girls, higher grades, prolonged exposure to screens, and higher perceived stress.
The authors add information regarding the relations between dry eye symptoms and perceived stress that was prevalent during the pandemic. In addition, they identified the daily cutoff exposure time to digital media devices, analyzed by odds ratio.
The discussion deals with the findings in relation to previous studies in the literature. The authors demonstrate a critical approach in the study limitations section, especially the lack of clinical evidence to dry eye. It is very relevant in teenagers' populations, which often demonstrate confounding results, especially regarding to self-reports of smoking in the formal educational system context.

*We are absolutely agres that the survey in formal educational system is a limitation of smoking factor, so we added this limitation as shown in lines 324 – 327.*

7. Conclusions
The conclusion repeats the study findings and lack the bottom-line importance and significant contribution of the study, as well as lessons learned and recommendations for the future.

*Thank you for recommendation. We have revised the conclusion section and provided the significant contribution of the study, as shown in lines 332 – 339..*

**Reviwer 2 Comments**

1. You mention “causing a negative impact on ocular surface” but the reference you bring showed only change in OSDI and not the other Ocular surface findings which showed no correlation.
*Thank you for recommendation. We deeply agree that OSDI is not associated with the ocular surface findings. Thus, we have removed this reference.*

*We added the study of “Blinking and normal ocular surface in school-aged children and the effect of age and screen time”.^4^ This study suggested that screen time may be associated with the ocular surface (Tear meniscus height (TMH) and non-invasive tear break-up time (NIBUT)).*

*We have also added the study of “Ocular surface predisposing factors for digital display-induced dry eye”.^5^ This study suggested that digital screen tasks alter the TMH and NIBUT.*

2. Line 69-70 you write about increased screen exposure and also mention social media – after reading the reference don’t see how’s it all related. Please elaborate or delete or change reference.

*Thank you for recommendation. In the current version, this reference has been deleted.*

3. Line 120 you mention “blind” do you mean VA of NLP both eyes?

Yes, we referred to NLP in both eyes. To clarify this, we have revised the manuscript, as shown in lines 117 – 118.

4. You told the students about dry eye and then asked them questions about dry eye - Did you have any concerns about that issue? (when you mention something and then ask about it – it might raise awareness)

*Thank you for pointing this out. We are concerned about satisficing (respondents’ answers to the questionnaire are of low quality).^6^ Most Thai students don’t know about dry eye. Thus, we tried to motivate the guardians and students to respond to the questionnaire by giving them information about dry eye.*

*We agree with you that providing the information may raise awareness. However, awareness-raising requires multiple processes for successful implementation.^7^*

5. If you had almost 70% Females and DED was more prevalent among females – don’t it change all the statistics? Could you elaborate?

*Thank you for your question. Thus, we stratified the analysis of the female group. The results of significant association are still the same in both the univariate and multivariate analyses.*

**References**

1.	Garcia-Ayuso D, Di Pierdomenico J, Moya-Rodriguez E, Valiente-Soriano FJ, Galindo-Romero C, Sobrado-Calvo P. Assessment of dry eye symptoms among university students during the COVID-19 pandemic. Clin Exp Optom. 2021:1-7.

2.	Tangmonkongvoragul C, Chokesuwattanaskul S, Khankaeo C, Punyasevee R, Nakkara L, Moolsan S, et al. Prevalence of symptomatic dry eye disease with associated risk factors among medical students at Chiang Mai University due to increased screen time and stress during COVID-19 pandemic. PLoS One. 2022;17(3):e0265733.

3.	Lin F, Cai Y, Fei X, Wang Y, Zhou M, Liu Y. Prevalence of dry eye disease among Chinese high school students during the COVID-19 outbreak. BMC Ophthalmol. 2022;22(1):190.

4.	Chidi-Egboka NC, Jalbert I, Wagner P, Golebiowski B. Blinking and normal ocular surface in school-aged children and the effects of age and screen time. Br J Ophthalmol. 2022.

5.	Talens-Estarelles C, Garcia-Marques JV, Cervino A, Garcia-Lazaro S. Ocular surface predisposing factors for digital display-induced dry eye. Clin Exp Optom. 2022:1-7.

6.	Krosnick JA. Response strategies for coping with the cognitive demands of attitude measures in surveys. 1991;5(3):213-36.

7.	Sayers R. Principles of awareness-raising for information literacy: a case study. Bangkok: Communication and Information (CI) UNESCO Asia and Pacific Regional Bureau for Education 2006. 124 p.
