# Supplementary material for: Prevalence and associated factors for self-reported symptoms of dry eye among Thai school children during the COVID-19 outbreak
Source: PLoS One. 2023 Apr 24;18(4):e0284928. doi: 10.1371/journal.pone.0284928 (PMC10124893; doi:10.1371/journal.pone.0284928)
Supplement: Attachment — Submitted filename: Response_to_Reviewers.docx [file pone.0284928.s003.docx]

**Journal Requirements:**

Please review your reference list to ensure that it is complete and correct. If you have cited papers that have been retracted, please include the rationale for doing so in the manuscript text, or remove these references and replace them with relevant current references. Any changes to the reference list should be mentioned in the rebuttal letter that accompanies your revised manuscript. If you need to cite a retracted article, indicate the article’s retracted status in the References list and also include a citation and full reference for the retraction notice.

*Thank you for your message. In the revised version, we checked all the references and ensured that they are compleate and correct.*

**Reviewer #1:**

**General comment:** The authors of the manuscript have definitely shown improvement in the structure, and the improvement is noticeable throughout the entire manuscript. The authors have addressed all previous comments. The sequence of the paper is better organized. The literature review is more robust and up to date. The method and results sections are much clearer and transparent. The authors use the added references in the discussion, making it easier for the reader to understand how the work they have done contributes to current scientific literature.
I now find the manuscript publishable, however I suggest four additional minor revisions.

1. Line 71: The authors mentioned the importance of TFOS epidemiologic report. I recommend adding a few words to summarize its main conclusions rather than its call for additional research.

*Thank you for you recommendation. We revised this statement and added the summary from the TFOS epidemiology report (line 72-74).*

2. Line 117: Lack of light perception, the precise professional definition is "No light perception".

*Thank you for your suggestion. In revised manuscript, we used “no light perception” (line 118).*

3. Line 254, should read: There are no validated questionnaires for "dry eye symptoms", or cut off scores.
*Thank you for pointing this out; we revised this statement (line 254)*

4. The paragraph starting from 277: The authors added important comparison with other dry eye studies which used different questionnaires. The reader can benefit from some more critical information regarding the different or similar parameters those questionnaires used.

*Thank you for your suggestion. We revised this paragraph and provided details of these questionnaires. We believe that the reader will benefit from this section (in lines 280-294).*

Reviewer #2:

Thanks for reading, understanding and integrating our comments. I think this new paper much better and ready for publication.

*We appreciate your kind comment and thank you for reviewing our manuscript.*
